# Prenylated Polyphenols from *Broussonetia kazinoki* as Inhibitors of Nitric Oxide Production

**DOI:** 10.3390/molecules23030639

**Published:** 2018-03-12

**Authors:** Da Yeon Lee, Hwa Jin Lee, Jae-Ha Ryu

**Affiliations:** 1 College of Pharmacy and Research Center for Cell Fate Control, Sookmyung Women’s University, Seoul 04310, Korea; ldy38@naver.com; 2 School of Industrial Bio-Pharmaceutical Science, Semyung University, Jecheon, Chungbuk 27136, Korea; hwalee@semyung.ac.kr

**Keywords:** *Broussonetia kazinoki*, prenylated polyphenols, nitric oxide, inducible nitric oxide synthase, nuclear factor κB

## Abstract

Excessive nitric oxide (NO) production by macrophages has been involved in inflammatory diseases. Seven polyphenols (**1**–**7**) were isolated from *Broussonetia kazinoki (B. kazinoki)* and investigated as potential inhibitors of NO overproduction in lipopolysaccharide (LPS)-activated RAW 264.7 cells. Among them, four prenylated polyphenols (**2**–**4** and **6**) with a catechol moiety efficiently suppressed the LPS-induced high level of NO with IC_50_ values of less than 6 µM. The compounds **2**–**4** and **6** also attenuated protein and mRNA levels of inducible nitric oxide synthase (iNOS). Moreover, they suppressed the nuclear factor κB (NF-κB) activity by inhibiting the degradation of inhibitory-κB-α (I-κB-α) and the translocation of NF-κB into the nucleus in LPS-activated macrophages. Taken together, these findings suggest that polyphenols from *B. kazinoki* might be beneficial for treatment of inflammatory diseases.

## 1. Introduction

*Broussonetia kazinoki* (*B. kazinoki*), called as paper mulberry, has been used as a diuretic, a tonic, and a suppressant of edema in Chinses folk medicine. *B. kazinoki* has been reported to have various biological activities including anti-atopic dermatitis [1], anticancer [2], anti-inflammation [3], and depigmenting effects [4]. We have reported several prenylated polyphenols from *B. kazinoki* such as phytoestrogen [5], Fyn kinase inhibitors [6], ERK inhibitors [7], AMPK activators [8], and stimulators of myoblast differentiation [9]. In addition, kazinol B, a prenylated flavan with a dimethyl pyrane ring, was reported as an inhibitor of nitric oxide production without clarification of the molecular mechanism [3]. Nitric oxide (NO) is an unstable gas synthesized from an l-arginine substrate via catalytic reaction by nitric oxide synthase (NOS) [10]. The inducible NOS (iNOS) is primarily found in macrophages and induced by lipopolysaccharide (LPS) or cytokines to produce a high level of NO as a pro-inflammatory mediator [11]. A large amount of NO reacts with superoxide anion (O_2_^•−^) to generate peroxynitrite (ONOO^−^), an extremely reactive nitrogen species [12]. Consequently, the NO/iNOS pathway has been involved in inflammatory condition such as atherosclerosis [13], allergic pulmonary inflammation [14], diabetes [15], Alzheimer’s disease [16], and cancer [17]. Therefore, the inhibition of excessive NO production can be a critical strategy for the treatment of inflammatory diseases. This study shows that the bioactive polyphenols from *B. kazinoki* extract have a suppressive effect on NO production in LPS-activated macrophages and reveal the working mechanism for these polyphenols’ activities.

## 2. Results and Discussion

**Figure 1:**
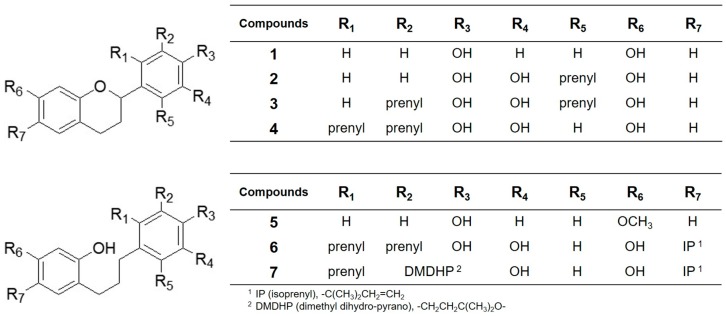
The chemical structures of Compounds **1**–**7** isolated from *Broussonetia kazinoki*.

In our effort to search for the plant-derived inhibitors of NO production, we observed the EtOAc soluble layer of *Broussonetia kazinoki* extract exerted potent inhibitory activity of NO production in LPS-activated macrophages. Activity-guided separations from *B. kazinoki* yielded seven polyphenols (**1**–**7**) whose chemical structures were elucidated as tupichinol C (**1**) [18], kazinol U (**2**) [5], kazinol A (**3**) [19], kazinol I (**4**) [20], broussonin A (**5**) [21], kazinol C (**6**) [20], and kazinol D (**7**) [20] (Figure 1) by analyzing the spectroscopic materials and verified by comparison with previously described data.

These seven polyphenols (**1**–**7**) demonstrated a concentration-dependent inhibition of NO production in LPS-stimulated RAW 264.7 macrophages (0–20 µM). Their IC_50_ values are presented in Table 1.

**Figure 2:**
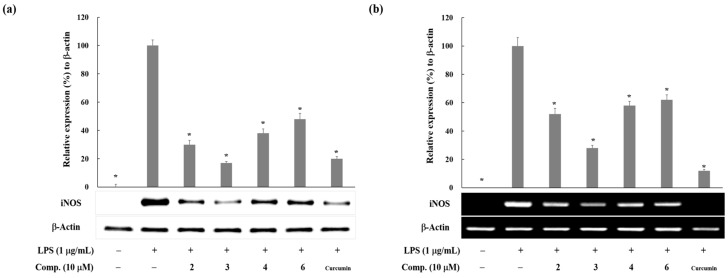
The inhibitory effects of Compounds **2**–**4** and **6** from *B. kazinoki* on LPS-induced iNOS expressions. (**a**) Effect of Compounds **2**–**4** and **6** on iNOS protein levels in LPS-activated RAW 264.7 cells. iNOS and β-actin protein levels were determined by Western blotting. The relative intensity of iNOS to β-actin bands was measured by densitometry. The values express the means ± S.D. of three experiments. * *p* < 0.05; (**b**) Effect of Compounds **2**–**4** and **6** on iNOS mRNA levels in LPS-activated RAW 264.7 cells. The mRNA levels of iNOS and β-actin were determined by RT-PCR. The relative intensity of iNOS to β-actin bands was measured by densitometry. The values express the means ± S.D. of three experiments. * *p* < 0.05. Curcumin was used as the positive control. Images are the representative of three independent experiments with similar results.

Compounds **1**–**7** concentration-dependently inhibited the LPS-induced NO production (IC_50_ = 4.2 ~ >20 µM) and the activity was affected by their chemical structures. IC_50_ values of the prenylated Compounds **2**–**4**, **6**, and **7** were less than 10 µM, while those of the non-prenylated Compounds **1** and **5** were 16.9 and higher than 20 µM, respectively. These findings are in agreement with the previously reported result that prenylated polyphenols, compared with non-prenylated polyphenols, showed higher potency in antioxidant and cyto-protective activity against oxidative stress [6]. These might come from the increased hydrophobicity and cell permeability of prenylated compounds [6]. Among prenylated polyphenols, Compounds **2**–**4** and **6** showed a more potent inhibition of NO production (IC_50_ values: 4.2~5.3 µM) than Compound **7** (IC_50_ value: 8.0 µM). Compounds **2**–**4** and **6** have a catechol moiety in prenylated aromatic rings, while Compound **7** has a dimethyl dihydropyrane ring that was formed by an oxidative cyclization of one prenyl chain with the hydroxyl group of Compound **6**. Based on these observations, we postulated that prenyl groups and a catechol moiety in an aromatic ring are favorable structures for the inhibitory activity of NO production. Next, we investigated protein levels of iNOS by Western blot analysis to disclose the underlying mechanism of Compounds **2**–**4** and **6** for the inhibition of LPS-induced NO production. As shown in Figure 2a, they (10 µM) suppressed the LPS-activated iNOS protein expression in RAW 264.7 cells. Curcumin was used as a positive control for the level of iNOS expression. The inhibitory effect of Compound **3** on iNOS protein expression was similar with that of curcumin, while other compounds were weaker than curcumin. Furthermore, they attenuated the levels of iNOS mRNA in LPS-activated RAW 264.7 cells (Figure 2b). These results reveal that Compounds **2**–**4** and **6** suppress the LPS-activated iNOS expression at the transcriptional level.

**Figure 3:**
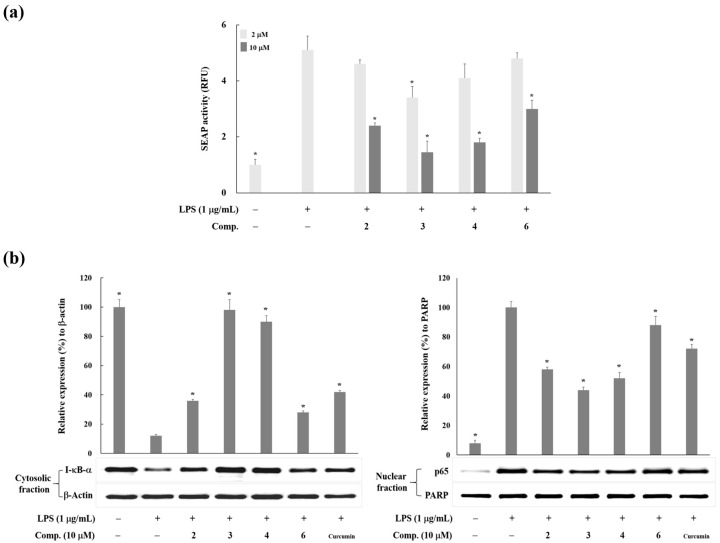
The inhibitory effects of Compounds **2**–**4** and **6** from *B. kazinoki* on LPS-induced NF-κB activity and nuclear accumulation. (**a**) Effect of Compounds **2**–**4** and **6** on LPS-induced NF-κB transcriptional activation in stably transfected RAW 264.7 cells with pNF-κB-SEAP-NPT (T-RAW 264.7 cells). Cells were pretreated with Compounds **2**–**4** and **6** for 30 min prior to LPS treatment (1 µg/mL) for 6 h. Aliquots of culture medium were analyzed for the measurement of secretory alkaline phosphatase (SEAP) activity. The values present as means ± S.D. of three independent experiments. * *p* < 0.05; (**b**) The effect of Compounds **2**–**4** and **6** on the degradation of I-κB-α and the accumulation of p65 in nuclei in LPS-stimulated macrophages. RAW 264.7 cells were pretreated with compounds for 30 min prior to LPS treatment (1 µg/mL) for 15 min. Cytosolic and nuclear extracts were prepared for the Western blotting of I-κB-α and p65, respectively. The relative intensities of I-κB-α to β-actin and p65 to PARP bands were measured by densitometry. The values express the means ± S.D. of three experiments. * *p* < 0.05. Curcumin was used as the positive control. Images are the representative of three independent experiments with similar results.

Since iNOS gene expression is mainly regulated at the transcriptional level by nuclear factor κB (NF-κB) [22], we examined the transcriptional activity of NF-κB using T-RAW 264.7 macrophage cells, which were stably transfected RAW 264.7 cells with NF-κB-SEAP-NPT reporter construct [23]. SEAP activity was increased by LPS treatment, which means the activation of NF-κB in stimulated macrophages (Figure 3a). Compounds **2**–**4** and **6** downregulated the LPS-induced NF-κB activation in T-RAW 264.7 cells. This result suggests that Compounds **2**–**4** and **6** suppressed NF-κB activation in LPS-stimulated T-RAW 264.7 cells. In addition, NF-κB, a pivotal regulator in the inflammatory response, is present in the cytoplasm as an inert dimer (the p50 and p65 subunits), complexed with an inhibitory protein I-κB-α in a normal condition. Via pro-inflammatory stimuli, phosphorylation of I-κB-α occurs, and phosphorylated I-κB-α is quickly degraded by the proteasome, leading to the dissociation of NF-κB from I-κB-α and translocation of NF-κB (p50/p65 dimer) into the nucleus [24]. The active NF-κB binds to the specific DNA sequence of target gene and regulates the transcription of inflammatory genes including iNOS. Herein, we investigated whether Compounds **2**–**4** and **6** affect the degradation of I-κB-α in cytoplasm and the translocation of NF-κB into the nucleus. Compounds **2**–**4** and **6** decreased the cytoplasmic I-κB-α degradation and suppressed the level of nuclear p65 in LPS-activated macrophages (Figure 3b). These observations indicate that these prenylated polyphenols (**2**–**4** and **6**) stabilized I-κB-α and prevented the nuclear translocation of the p65 subunit of NF-κB.

## 3. Materials and Methods

### 3.1. Plant Material, Extraction, Isolation, and Sample Preparation

The air-dried root bark of *Broussonetia kazinoki* (0.6 kg), collected from Goesan, Chungbuk province, Korea in 2014 (voucher specimen no. SPH 1401), was extracted for 24 h at room temperature with 2 L of ethanol. The extract (51 g) was suspended in water and successively partitioned with *n*-hexane, EtOAc, CHCl_3_, and BuOH. The EtOAc soluble layer (31 g) was subjected to silica gel column chromatography eluting with *n*-hexane/acetone (20:1→1:10) to collect 11 fractions. Fraction 5 was separated on silica gel using *n*-hexane/EtOAc gradient system (20:1→1:3) to yield eleven subfractions. Subfraction 5.7 was chromatographed on an RP-C18 column with elution system of MeOH/water (50%→100%), and then it’s Subfraction 5.7.4 was re-chromatographed on an RP-C18 column with MeOH/water gradient elution (50%→100%) to yield kazinol D (**7**, 60 mg). Fraction 6 was chromatographed on a silica gel column with *n*-hexane/acetone (40:1→1:1) to yield six subfractions. Subfraction 6.4 was further chromatographed on a silica gel column using *n*-hexane/EtOAc (20:1→2:1) to afford kazinol A (**3**, 23 mg) and Subfraction 6.6 was chromatographed on an RP-C18 column with elution system of MeOH/water (30%→100%) to afford kazinol I (**4**, 79.8 mg). Fraction 7 was further chromatographed with a silica gel column with CHCl_3_/MeOH (100:1→1:1) to afford six subfractions. Fraction 7.3 was chromatographed on an RP-C18 column with MeOH/water (40%→100%) to yield broussonin A (**5**, 17 mg), tupichinol C (**1**, 7 mg), and kazinol C (**6**, 260 mg). Fraction 8 was chromatographed on an RP-C18 column with MeOH/water (30%→100%) to afford kazinol U (**2**, 139 mg). Test materials were dissolved in dimethyl sulfoxide (DMSO), and the final concentration of DMSO in cell culture media was less than 0.1%, which does not affect the cell viability or other cellular functions.

### 3.2. Cell Culture

A murine macrophage cell line, RAW 264.7 cells (ATCC, Rockville, MD, USA) were cultured in DMEM containing 10% FBS, 100 units/mL penicillin, and 100 µg/mL streptomycin (Life Technologies, Frederick, MD, USA). Cells were incubated at 37 °C with 5% CO_2_ in a humidified atmosphere.

### 3.3. Measurement of Nitric Oxide Production and Cell Viability

RAW 264.7 cells (1 × 10^5^ cells/48 well plate) were treated with or without compounds in presence of LPS (1 µg/mL) for 20 h. NO was measured by detecting the accumulated nitrite by the Griess method. Briefly, aliquots (100 µL) of culture media were mixed with 150 µL of Griess reagent (1% sulfanilamide, 0.1% naphthylethylene diamine in 2.5% phosphoric acid solution) and then waited at room temperature for 10 min in a 96-well microplate. Absorbance was detected at 540 nm by using a microplate reader (Molecular Devices, Sunnyvale, CA, USA). The concentration of NO was assessed by the sodium nitrite standard curve. To evaluate cell viability, cells were plated at a density of 1 × 10^3^ cells/well in a 96-well plate. The cells were incubated with LPS (1 µg/mL) in the presence of Compounds **2**–**4** and **6** (10–20 µM). After 20 h, cells were treated with 3-(4,5-dimethylthiazol-2-yl)-2,5-diphenyltetrazolium bromide (MTT, 5 mg/mL) for 4 h and then added the DMSO to solubilize the formazan. Absorbance was read at 540 nm by using a microplate reader (Figure S1).

### 3.4. Western Blot Analysis

RAW 264.7 cells (5 × 10^5^ cells/60 mm dish) were treated with or without test compounds in the presence of LPS (1 μg/mL). After 20 h treatment, cells were lysed with cell lysis buffer (Cell Signaling Technologies, Beverly, MA, USA) and centrifuged at 4 °C. Supernatants were subjected to the quantitation of protein concentrations by the Bradford method. For preparation of cytosol and nuclear extracts, cells were treated with test compounds for 30 min prior to stimulation with 1 μg/mL LPS. Following 15 min treatment of LPS, cells were collected using NE-PER nuclear and cytoplasmic extraction reagents according to the manufacturer’s instructions (Pierce Biotechnology, Rockford, IL, USA). The lysates were resolved by SDS-PAGE and transferred to a PVDF membrane. The membrane was incubated with 5% nonfat milk for 1 h and then incubated with the primary antibody overnight at 4 °C. For immunoblot analysis, antibodies against iNOS, I-κB-α, and p65 were obtained from BD Biosciences (Franklin Lakes, NJ, USA) and Santa Cruz Biotechnology (Rockford, IL, USA), respectively. After incubation with the secondary antibody for 1 h at room temperature, bands were detected by VersaDoc 3000 (Bio-Rad, Hercules, CA, USA) with ECL reagents (GE Health Care Life Sciences, Marlborough, MA, USA).

### 3.5. Reverse Transcription and Polymerase Chain Reaction (RT-PCR) Analysis

RAW 264.7 cells (5 × 10^5^ cells/60 mm dish) were stimulated for 6 h with or without test compounds in the presence of LPS (1 μg/mL). Total RNA was extracted by TRIzol (Life technologies, Frederick, MD, USA) according to the manufacturer’s instructions and then reverse transcribed into cDNA using reverse transcriptase (Life Technologies, Frederick, MD, USA) and random hexamer (Cosmo, Seoul, Korea). Then, prepared aliquots of cDNA were amplified using a recombinant Taq polymerase (Promega, Madison, WI, USA) and primers for iNOS and β-actin to detect gene expression of iNOS and β-actin.

### 3.6. Measurement of NF-κB Activity

NF-κB transcriptional activity was assessed using the stably transfected RAW 264.7 cells with pNF-κB-SEAP-NPT (T-RAW 264.7 cells were kindly gifted by Professor Yeong Shik Kim, Seoul National University, Seoul, Korea) as described previously, with some modifications [23]. Briefly, T-RAW 264.7 cells were plated on a 24-well plate overnight. Test compounds were added to cells 30 min before the treatment with LPS (1 µg/mL). After 6 h of incubation, aliquots of culture medium were heated at 65 °C for 6 min, and the activity of SEAP (secretory alkaline phosphatase) was then evaluated. The transcriptional activity was presented as relative fluorescence units (RFUs).

### 3.7. Statistical Analysis

The values express mean ± S.D. of three experiments, and statistical analysis was carried out with a one-way ANOVA and Student’s *t*-test. A *p*-value of <0.05 was considered significantly different.

## 4. Conclusions

Our results indicate that seven polyphenols (1–7) from *B. kazinoki* inhibited NO production in LPS-stimulated RAW 264.7 cells. Four prenylated polyphenols (2–4 and 6) with a catechol moiety in an aromatic ring potently suppressed NO production, iNOS protein/mRNA expression, and NF-κB activity. They also suppressed the degradation of I-κB-α and the nuclear translocation of NF-κB in LPS-stimulated RAW 264.7 macrophages. The active compounds, kazinol U (2), kazinol A (3), kazinol I (4), and kazinol C (6), from *B. kazinoki* have potential for use in the treatment of diseases concomitant with NO overproduction.

## Figures and Tables

**Table 1:** The suppressive effect of Compounds **1**–**7** from *B. kazinoki* on NO production in LPS-activated RAW 264.7 macrophages.

Compounds	IC_50_ (µM) ^1^
1	>20
2	5.2 ± 0.2
3	4.2 ± 0.2
4	5.3 ± 0.4
5	16.9 ± 1.1
6	5.0 ± 0.4
7	8.0 ± 0.5

^1^ IC_50_, 50% inhibitory concentration. The values are means ± S.D. of three experiments.

## Supplementary Materials

The supplementary materials are available online.
