# Supplementary material for: Prenylated Polyphenols from Broussonetia kazinoki as Inhibitors of Nitric Oxide Production
Source: Molecules. 2018 Mar 12;23(3):639. doi: 10.3390/molecules23030639 (PMC6017281; doi:10.3390/molecules23030639)
Supplement: Supplementary file 1 [file molecules-23-00639-s001.pdf]

Supplementary Materials

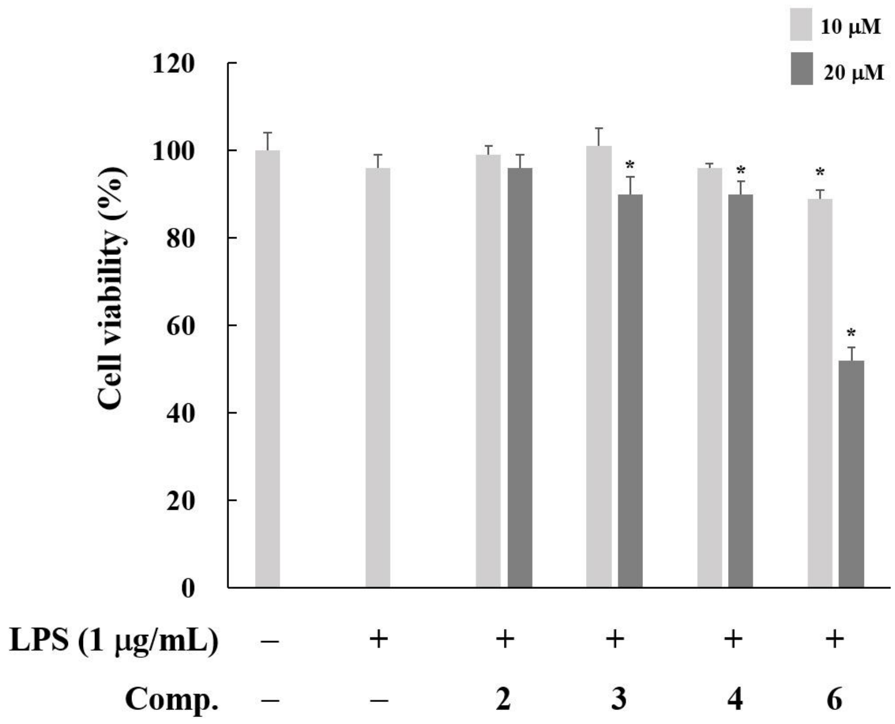

Figure S1. The effect of compounds **2-4** and **6** on cell viability of RAW 264.7 macrophages. Cells were treated with LPS (1 µg/mL) in presence or absence of test compounds (10 and 20 µM). Cell viability was determined by MTT assay. The values are expressed as the means ± S.D. of three experiments. * $p < 0.05$ (*vs*. vehicle control).
